# STRP: A Low-Cost Teleoperation Platform with Spatial Force Feedback for Visually Occluded Dexterous Manipulation in Hazardous Environments

**DOI:** 10.3390/biomimetics11090609

**Published:** 2026-08-28

**Authors:** Jingyuan Luo, Siquan Wu, Li Shen, Chuliang Chi, Hao Wu, Yongquan Chen

**Affiliations:** 1 Shenzhen Institute of Artificial Intelligence and Robotics for Society, Longgang District, Shenzhen 518129, China; luojingyuan1@mails.gdut.edu.cn (J.L.); 225040259@link.cuhk.edu.cn (S.W.); 222010062@link.cuhk.edu.cn (C.C.); 2 School of Science and Engineering, The Chinese University of Hong Kong, Longgang District, Shenzhen 518172, China; 3 Shenzhen XJCSENSOR Technology Co., Ltd., Shenzhen 518115, China; rdc@xjcsensor.com (L.S.); info@xjcsensor.com (H.W.)

**Keywords:** teleoperation, haptic feedback, exoskeleton glove, six-axis force/torque sensor, dexterous manipulation

## Abstract

High-voltage grid maintenance and chemical processing demand dexterous manipulation in confined, visually occluded environments. Existing teleoperation systems present a severe cost–fidelity trade-off: industrial platforms exceed USD 60,000 yet lack spatial directional resolution, while affordable alternatives omit fingertip force sensing entirely. We present STRP (Spatial Teleoperation Research Platform), a low-cost teleoperation system with concurrent motion capture and directional haptic feedback that encodes fingertip six-axis force vectors into four-directional vibrotactile cues (left, right, dorsal, volar). The master interface is a wearable 15-DoF exoskeleton glove with Hall-effect joint sensing and a 16-channel LRA array; the slave end is a 17-DoF dexterous hand with integrated micro six-axis force/torque sensors. Experimental validation demonstrates 97.5% blind discrimination accuracy for directional cues and an end-to-end latency of approximately 28 ms. In teleoperated pick-and-place tasks, multi-directional tactile feedback reduced task completion time by 41% (31.2 s to 18.4 s) and peak grip force by 32% (4.11 N to 2.79 N) versus no-feedback baselines. In visually occluded wall exploration, directional feedback enabled systematic location of all target walls, whereas single-site volar feedback permitted detection of only volar-facing surfaces and no-feedback conditions precluded any spatial reasoning. These results identify spatial directional vibrotactile feedback as a distinct sensory channel that cannot be substituted by magnitude-only vibration or vision alone. STRP establishes a foundation for affordable, high-fidelity haptic teleoperation in hazardous environments.

## 1. Introduction

High-voltage grid maintenance, chemical processing in fume hoods, and nuclear decommissioning require high dexterity and judgment for operations within confined, hazardous environments [1,2,3]. These tasks demand fine force control and tactile sensitivity to manipulate energized components, fragile glassware, or contaminated materials, while metal enclosures and opaque containers often obstruct visual line-of-sight, creating visually occluded conditions where traditional vision-based manipulation fails [4,5]. Teleoperation systems that enable remote robotic control while maintaining haptic awareness are therefore essential for ensuring both safety and operational effectiveness in these occluded scenarios.

Beyond mere contact detection, these tasks require spatial directional perception at the fingertip: the operator must discern not only how hard an object is being touched, but from which direction the contact originates—left, right, dorsal, or volar [6]. Without this directional channel, operators cannot perform exploratory motions or adjust grasp strategies in the absence of vision, leading to excessive grip forces, high collision rates, and damage to fragile equipment.

Despite decades of research, existing teleoperation solutions present a stark trade-off between affordability and tactile fidelity that leaves this directional perception gap unaddressed. High-end industrial systems, such as the Shadow Dexterous Hand paired with kinesthetic exoskeletons (e.g., HaptX), provide rich whole-hand force feedback but cost exceeding USD 60,000 [7,8]. A critical limitation of these actuated kinesthetic exoskeletons is their lack of spatial directional resolution: they can convey force magnitude through joint resistance, yet cannot discriminate whether contact occurs on the left, right, dorsal, or volar aspect of the fingertip [9,10]. Commercial wearable master interfaces—including data gloves (Manus, SenseGlove) and force-feedback exoskeletons (HexoTrac, Dexta)—have advanced motion capture and kinesthetic rendering [11,12,13]. Sensorized and soft exoskeleton gloves have likewise matured in tracking accuracy and wearability [14,15,16,17], yet their feedback modality remains predominantly non-directional. Consequently, operators must infer contact direction visually, which fails under occlusion.

At the sensory level, miniaturized six-axis force/torque sensors have enabled fingertip force and torque measurement for robotic hands [18], yet the rendering of this information to the human operator remains problematic. Haptic feedback modalities are broadly divided into kinesthetic (force reflected at the joints) and vibrotactile (vibratory stimuli on the skin surface) [7,9]. Kinesthetic feedback excels at conveying load magnitude but requires bulky actuators; vibrotactile feedback—particularly via linear resonant actuators (LRAs)—offers a lightweight alternative that can be spatially distributed around the fingertip to encode direction. Recent fingertip tactile sensors such as GelSight and 9DTact have demonstrated high-resolution geometry and force estimation [19,20], but their integration with spatially resolved vibrotactile displays for real-time teleoperation remains underdeveloped in low-cost systems.

In contrast, affordable alternatives such as vision-based systems (e.g., LEAP Hand, AnyTeleop) and simplified data gloves cost less than USD 2000 but omit fingertip force sensing entirely, leaving operators effectively “blind” to contact location and magnitude [4,21]. This sensory deficit is particularly dangerous in visually occluded manipulation, where exploratory motions reduce to inefficient trial-and-error search [5]. Recent low-cost open-source teleoperation systems have made significant strides in making dexterous manipulation more accessible by combining motion capture with haptic feedback: Aloha 2, Mobile Aloha, and UMI introduced low-cost hardware for bimanual data collection [22,23,24]; DexCap enabled scalable portable motion capture without gloves [25]; Open-Television and Bunny-VisionPro leveraged VR devices for immersive visual feedback [26,27]; AirExo demonstrated whole-arm manipulation with lightweight exoskeletons [28]. HOMIE combined an isomorphic upper-body exoskeleton with Hall-sensor gloves for whole-body humanoid teleoperation [29]. In parallel, recent control- and perception-level advances have pushed the boundary of dexterous manipulation: robust bimanual humanoid control under servo constraints [30], category-level semantically-aware grasping [31], and low-latency human–machine control interfaces [32] have matured the execution and interface layers, which in turn raises the demand for high-fidelity human-in-the-loop interfaces. However, these systems primarily render force magnitude or single-point vibrotactile cues rather than spatially resolved directional information. DOGlove and CDF-Glove have further advanced low-cost haptic teleoperation by integrating force feedback gloves with dexterous hands, yet their tactile rendering remains confined to magnitude or single-site vibration without explicit directional encoding [33,34]. The current landscape thus presents a sharp dichotomy: high-end systems provide rich but non-directional force feedback at prohibitive cost and weight, while affordable alternatives sacrifice directional tactile sensing altogether.

To close this gap, we present STRP (Spatial Teleoperation Research Platform), a low-cost teleoperation system that closes both the motor command loop (master to slave) and the haptic feedback loop (slave to master) with directional force-to-vibrotactile encoding, designed specifically for visually occluded environments. STRP is specifically suited to hazardous deployment for two reasons. First, the feedback channel is purely tactile and therefore immune to smoke, dust, opaque enclosures, and lighting failure that disable vision-based teleoperation, while the lightweight master interface keeps the operator at a safe standoff distance from the hazardous workspace. Second, by distributing vibrotactile cues across the four anatomical aspects of the fingertip, STRP emulates the skin’s natural spatial sensitivity, conveying contact direction through the same channel the operator would rely on in direct manual handling and thereby reducing adaptation burden in high-stress occluded operations. The master interface is a wearable 15-DoF linkage-based exoskeleton glove that captures joint angles via radially magnetized Hall-effect sensors and renders spatial cues through a multi-channel LRA array distributed across the left, right, dorsal, and volar quadrants of each fingertip. At the slave end, a commercially available 17-DoF dexterous robotic hand (ZWHAND) serves as the motion execution platform, with custom micro six-axis force/torque sensors co-developed with the manufacturer integrated at the fingertips to measure contact force vectors ($F_{x}/F_{y}/F_{z}$). A directional encoding algorithm maps these force vectors in real time into four-directional tactile cues, enabling pose-independent exploration—operators perceive contact direction regardless of finger orientation, thereby supporting directional exploration and vision-free grasping in occluded or unknown environments. The master–slave loop communicates over an RS485/Modbus-RTU backbone with an end-to-end tactile latency of approximately 28 ms, well within the sub-100 ms budget commonly required for real-time teleoperation. Experimental validation demonstrates 97.5% blind discrimination accuracy for the four directional cues, confirming that pose-independent spatial perception is reliably achieved through the distributed LRA array. In teleoperated pick-and-place tasks, multi-directional tactile feedback reduced completion time by 41% (from 31.2 s to 18.4 s) and peak grip force by 32% (from 4.11 N to 2.79 N) relative to no-feedback baselines. In visually occluded wall exploration, directional feedback enabled systematic identification of all target walls regardless of finger pose, whereas single-site volar feedback only revealed volar-facing surfaces and no-feedback conditions precluded any spatial reasoning. At a fraction of the cost of industrial six-axis solutions, STRP makes high-fidelity spatial force feedback accessible to hazardous environment research. Code and hardware designs will be open-sourced upon publication.

## 2. Materials and Methods

### 2.1. Design Requirements and Approach

**Figure 1:**
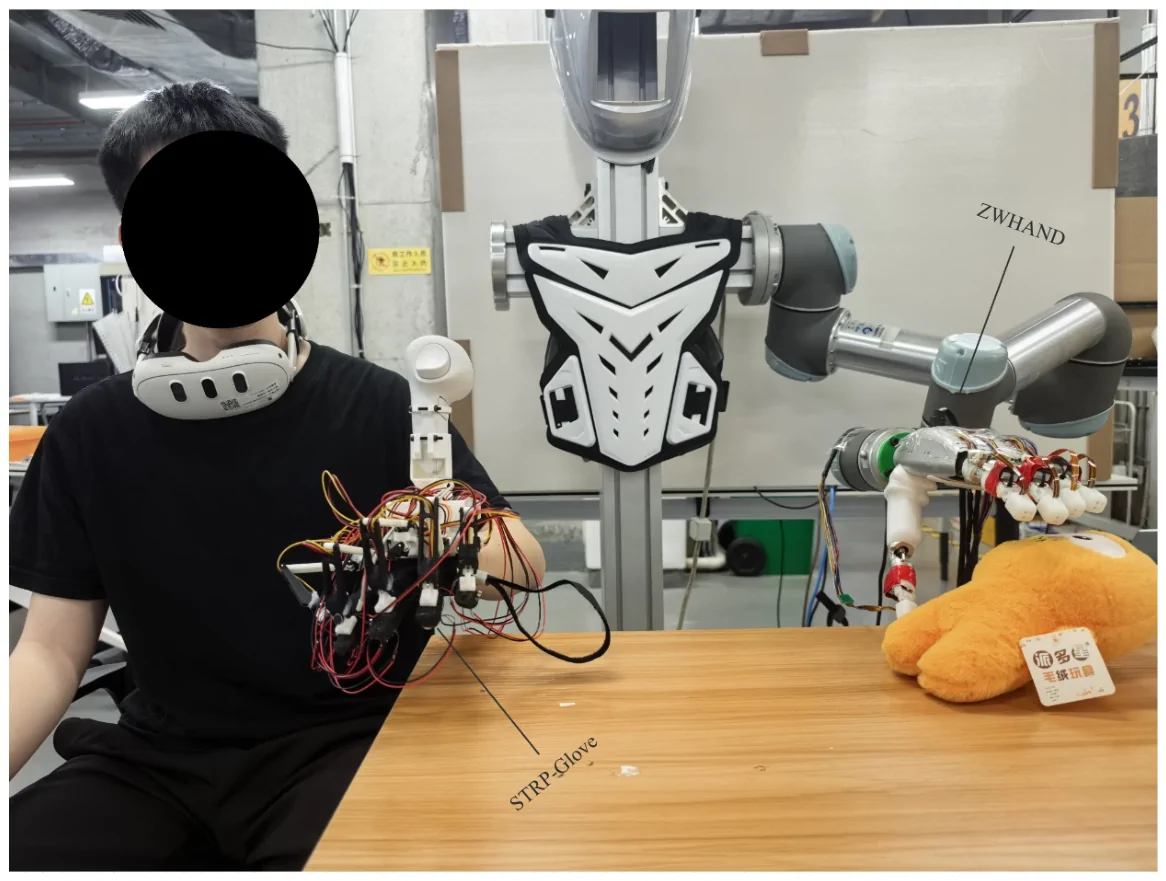
The STRP-Glove serves as the wearable master controller within the full teleoperation system. It is donned on the operator’s hand to acquire joint-angle data and concurrently render haptic feedback.

We present a multi-directional tactile feedback teleoperation system that closes the sensory loop between a master exoskeleton glove and a slave dexterous hand through micro fingertip six-axis force/torque sensing. Contact force vectors measured at the slave fingertips are mapped in real time to four-directional tactile cues (left, right, dorsal, and volar) on the operator’s skin, thereby enabling pose-independent exploration. This architecture enables accurate directional perception regardless of finger contact orientation, overcoming dexterous manipulation bottlenecks in visually occluded scenarios while enhancing teleoperation immersion and fine motor control. Figure 1 presents the physical prototype of the complete teleoperation system, comprising the STRP-Glove worn on the operator’s hand and a dexterous slave hand with integrated fingertip six-axis force/torque sensors mounted on a UR5 robotic arm.

To fulfill these design objectives, the master glove utilizes a linkage-based exoskeleton equipped with radially magnetized Hall-effect sensors to capture fifteen joint angles. By distributing compact linear resonant actuators (LRA) across the dorsal, volar, and lateral aspects of each fingertip, this configuration avoids the heavy motorized exoskeletons and bulky kinesthetic actuators characteristic of conventional force-feedback gloves. In addition, the low communication latency intrinsic to the dual-stage I^2^C-multiplexer haptic driver topology is indispensable for real-time tactile rendering. When anomalous force data are detected or the commanded vibration intensity exceeds comfortable physiological limits, the LRA drivers automatically idle their outputs to prevent sensory fatigue or perceptual misalignment.

The proposed system is configured as a master–slave teleoperation platform comprising a wearable exoskeleton glove (master) and a dexterous robotic hand (slave). The overall parameters of the STRP are first summarized in Table 1. The remainder of this section is then organized into two core subsystems: the master exoskeleton glove for motion capture and spatial tactile rendering (Section 2.2), and the slave fingertip six-axis force–torque sensing and integration (Section 2.3).

### 2.2. Master Interface: Exoskeleton Glove Design

#### 2.2.1. Measurement System

**Figure 2:**
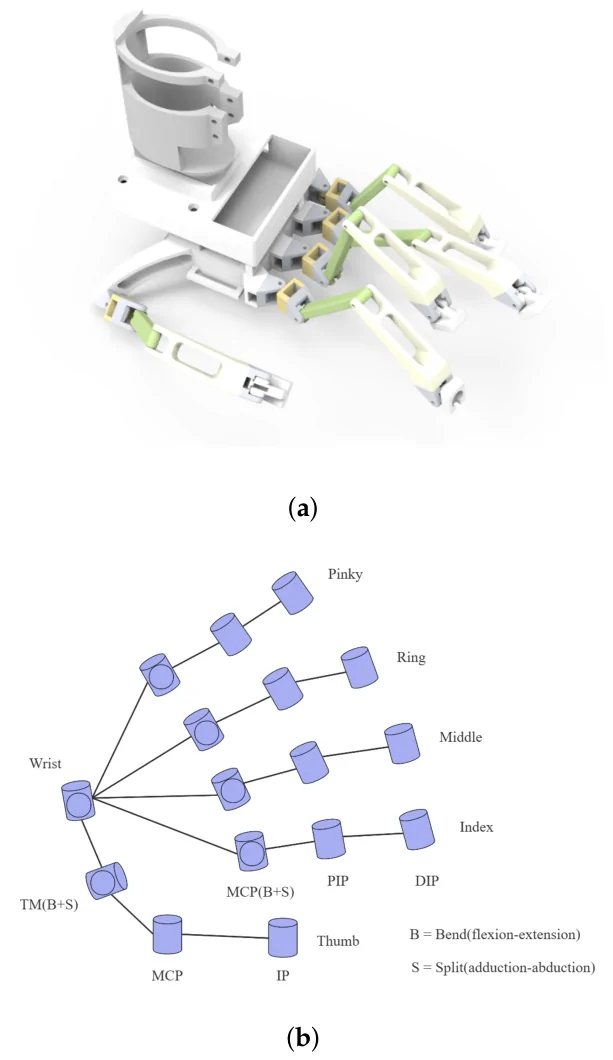
Overview of the STRP-Glove system. (**a**) Overall structural diagram of the STRP-Glove system. (**b**) Kinematic structure of the human hand system.

Prior work [35] has extensively characterized human hand kinematics. As depicted in Figure 2b, the skeletal structure primarily comprises hinge and ball-and-socket joints. The index, middle, ring, and pinky each contain two 1-DoF hinge joints—the distal interphalangeal (DIP) and proximal interphalangeal (PIP)—that enable flexion, whereas their metacarpophalangeal (MCP) joints possess two DoF, permitting both flexion and abduction–adduction. The thumb exhibits a distinct architecture: its interphalangeal (IP) and MCP joints are restricted to uniaxial bending, while the trapeziometacarpal (TM) joint operates as a saddle articulation allowing both bending and splaying. Additionally, the wrist contributes two DoF.

**Figure 3:**
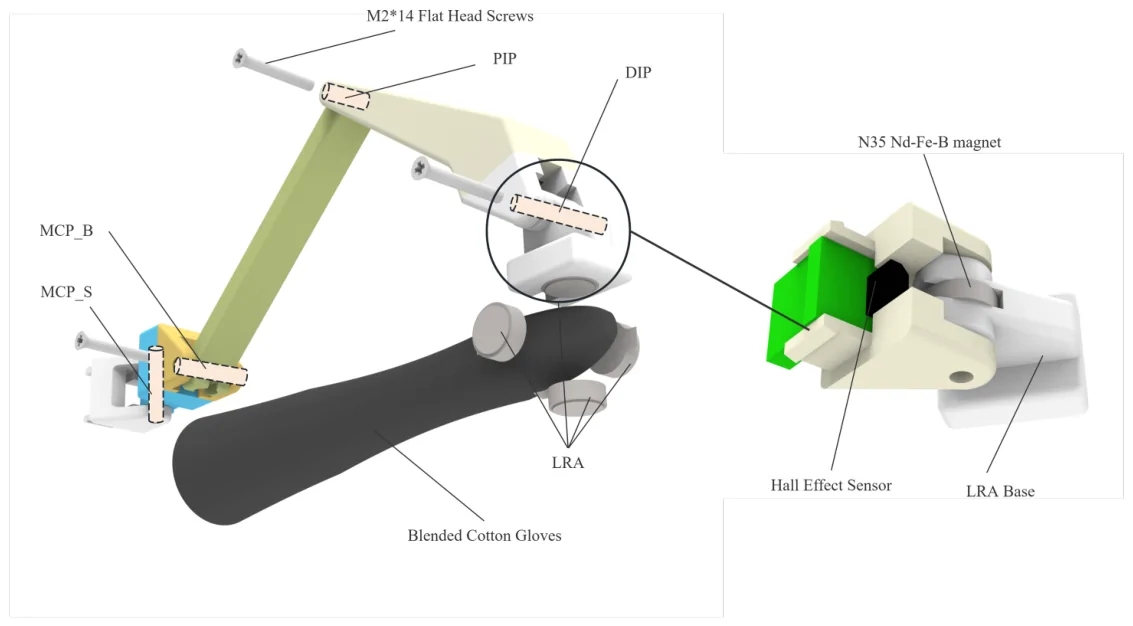
Exploded view of the single-finger structure. M2 × 14 denotes M2 flat-head screws of 14 mm length.

To measure the joint angles without encumbering the natural range of motion, the STRP-Glove employs a linkage-based mechanism instrumented with radially magnetized permanent magnets and three-dimensional Hall-effect sensors. As depicted in Figure 2 and detailed in the exploded view in Figure 3, each finger exoskeleton is primarily constructed from non-standard components 3D-printed from polylactic acid (PLA)—one of the most commonly used materials in exoskeleton manufacturing [36]—for a lightweight and low-cost design. The proximal link adjacent to the PIP joint measures 25 mm in length, whereas the distal link measures 45 mm. Adjacent phalangeal segments are connected by joint bases that form revolute pairs with the Hall-sensor mounting brackets.

The angle sensing unit comprises a radially magnetized neodymium-iron-boron (Nd-Fe-B) permanent magnet (grade N35, dimensions D7 × D2 × 2 mm) affixed to the rotating joint component, with its geometric center axis coincident with the joint rotation axis. The magnetization direction lies along the radial cross-section, such that the magnetic field vector rotates within the plane perpendicular to the joint axis. A single-axis linear Hall-effect sensor (model OH49E, sensitivity 2 mV/Gs, magnetic range ± 120 mT) is mounted tangentially on the side face of the magnet through a dedicated sensor bracket fixed to the adjacent link. This tangential placement ensures that the rotating radial magnetic field sweeps across the sensitive axis of the sensor, producing a scalar output proportional to the projection of the magnetic field onto that axis. Because the anatomical range of motion of the finger joint is limited (typically $<90^{{}^{\circ}}$), the sensor is mechanically aligned so that the entire working interval falls within a monotonic segment of the sinusoidal transfer characteristic. Consequently, the joint rotation angle can be unambiguously recovered from the scalar voltage via two-point calibration. This side-mounted configuration avoids occupying the axial space of the joint, which is critical for maintaining the compact, anthropomorphic profile of the exoskeleton glove.

Prior to operation, a 10 s calibration routine is executed in which the wearer repeatedly opens the hand fully and closes it into a fist. The system records the minimum and maximum magnetic flux densities corresponding to the fully extended and fully flexed postures, respectively. The measured magnetic range is then linearly mapped to the motor command output range of 0–1000 for the slave dexterous hand, with the instantaneous flux density scaled proportionally between the recorded extrema.

This per-session calibration routine is central to the robustness of the sensing scheme: by re-recording the magnetic extrema at the fully opened and fully closed postures before every use, the system absorbs slow variations arising from mechanical play and material creep into the recalibrated range, so that the mechanical structure and the calibration algorithm jointly maintain tracking consistency during operation.

The single-axis Hall sensor outputs a voltage proportional to the projection of the radial magnetic field onto its sensitive axis:

(1) $$V\left(\theta \right)=V_{\mathrm{offset}}+S\;B_{r}\cos\left(\theta -\theta_{0}\right),$$

where *S* denotes the sensor sensitivity (2 mV/Gs), $B_{r}$ is the radial magnetic flux density at the sensing location, and $\theta_{0}$ is the mechanical alignment offset between the sensitive axis and the magnet radial direction. Because the finger joint range $\Delta \theta <<90^{{}^{\circ}}$ is confined to a monotonic segment of the cosine characteristic, the angle is recovered unambiguously by linear mapping between the calibrated extrema:

(2) $$\theta =\theta_{\min}+\left(\theta_{\max}-\theta_{\min}\right)\;\frac{V-V_{\min}}{V_{\max}-V_{\min}}.$$

Here, $V_{\min}$ and $V_{\max}$ are the voltages recorded during the 10 s calibration routine (fully extended and fully flexed postures, respectively), and $\theta_{\min}$ and $\theta_{\max}$ are the corresponding joint limits.

Notably, the PIP and DIP joints are mechanically coupled; the PIP angle $\theta_{2}$ is derived from the measured DIP angle $\theta_{1}$ as shown in Equation (3) [37], thereby reducing the number of active sensors while preserving full-hand pose reconstruction.

(3) $$\theta_{2}=\frac{\theta_{1}+0.230}{0.989},$$

#### 2.2.2. Force Feedback System

Sixteen LD0825BC-0169F LRAs (Leader Micro Electronics (Huizhou) Co., Ltd., Huizhou, China) (8 mm × 2.55 mm) are mounted at the distal ends of the index, middle, ring, and pinky fingers, with four units per finger corresponding to the left, right, dorsal, and volar quadrants. The dorsal LRA is seated in a dedicated slot machined into the fingertip LRA base, while the remaining three LRAs are adhered to the outer surface of the glove. All LRAs are bonded to their mounting sites using circular 3M 9448HK cotton-paper double-sided tape; the LRA bases are subsequently bonded to the glove fabric with ergo5910 adhesive (viscosity 200–400 MPas) and mechanically secured to the exoskeleton Hall-sensor bases via M2 × 14 mm screws. The glove itself attaches to the exoskeleton through adhesive bonding at five fingertip points, with the palm section fastened to the underside of the exoskeleton base by hook-and-loop fasteners, allowing the wearer to adjust the fit according to hand size.

The miniature six-axis F/T sensor is rigidly mounted at the fingertip with its sensing axes aligned to the anatomical dorsal–volar and left–right directions through mechanical fixture design. Because the sensor frame is thereby coincident with the fingertip anatomy, no additional software coordinate transformation is required. Prior to each experimental session, a zero-offset calibration is executed while the hand remains relaxed and uncontacted: the system acquires 20 consecutive samples of $F_{x}$ and $F_{y}$ and averages them to obtain the bias terms $\left(b_{x},b_{y}\right)$. All subsequent readings are compensated by subtracting these biases:

(4) $$F_{x}=F_{x}^{\mathrm{raw}}-b_{x},\;F_{y}=F_{y}^{\mathrm{raw}}-b_{y}.$$

This calibration removes sensor drift and gravity-induced baseline offsets, ensuring that the directional decomposition responds only to external contact forces.

A hysteresis gate with a trigger threshold of 0.2 N and a release threshold of 0.1 N (50% ratio) suppresses rapid on–off chatter when the contact force hovers near the dead-zone boundary. The four LRAs on the contacting finger are driven simultaneously with independent intensities proportional to the absolute values of the x- and y-axis force components, thereby enabling the natural rendering of composite directional stimuli (e.g., dorsal–right). To achieve perceptually uniform force-to-vibration rendering, the system employs Stevens’ power law, a foundational psychophysical model stating that subjective sensation magnitude *S* grows as a power function of physical stimulus intensity *I* ($S=kI^{n}$) [38]. For vibrotactile stimulation of the glabrous fingertip mediated primarily by Pacinian corpuscles, reported power exponents range from 0.6 to 0.9 [39]. We select n=0.7 and set a mapping saturation threshold $F_{\max}=20.0$ N, with a 0.2 N dead zone to suppress sensor drift. The resulting mapping is given by Equation (5):

(5) $$V_{i}=g\left(A_{i}\right)=\left\{\begin{array}{cc} 0, & A_{i}<0.2\;N,\\ \left[127{\left(\frac{A_{i}}{20.0}\right)}^{0.7}\right], & 0.2\leq A_{i}<20.0\;N,\\ 127, & A_{i}\geq 20.0\;N, \end{array}\right.$$

### 2.3. Slave Device: Fingertip Force–Torque Sensing and Integration

#### 2.3.1. Dexterous Hand as the Experimental Platform

The slave manipulator employed in this study is a commercially available 17-DoF dexterous hand (ZWHAND, Shenzhen Zhaowei Machinery & Electronics Co., Ltd., Shenzhen, China). It provides 17 independent active degrees of freedom through embedded micro-stepper/hollow-cup motors coupled with ball-screw push-rod actuators. Each finger provides a flexion range of 90° at the metacarpophalangeal joint and 90° at the proximal interphalangeal joint, while the thumb provides 60° of opposition travel. The hand accepts position commands over RS485/Modbus-RTU at configurable baud rates of up to 2 Mbps, and its rated payload is 3 kg (firm grasp). The hand weighs 850 g (including wrist) and delivers a maximum grasping force of 30 N with individual fingertip contact forces in the range of 5–12 N. In the present work, the dexterous hand serves solely as the motion execution platform; the core contribution lies in the fingertip six-axis force–torque sensing subsystem and its closed-loop coupling with the master haptic interface.

At the system level, a host PC coordinates the bilateral master–slave teleoperation loop through two dedicated pathways: an ROS2-based control middleware handles the motion chain, while the tactile feedback chain runs as a dedicated sequential polling process. Joint angles captured by the STRP-Glove are transmitted to the host at 100 Hz via USB-UART, while six-axis force/torque feedback from the ZWHAND fingertips is returned over the RS485/Modbus-RTU bus at 100 Hz. The middleware integrates the glove and manipulator motion streams and generates synchronized motion commands for the UR5 arm (Ethernet, 100 Hz) and the ZWHAND (RS485/Modbus-RTU, 100 Hz), while the fingertip force/torque stream is acquired by the tactile polling process. The end-to-end latency from slave contact to master tactile rendering is approximately 28 ms, comprising the Modbus-RTU sensor readout (≈5 ms), host processing and USB-UART transmission (≈6 ms), the sequential I^2^C refresh of the four active channels of the contacting finger on the STM32 (≈8 ms), the DRV2605 register update (<1 ms), and a polling-phase scheduling margin of up to one host polling cycle (≈8 ms). The mechanical rise time of the LRA is intrinsic to the actuator physics, is common to all vibrotactile systems of this class, and is therefore not included in the communication latency above.

This architecture constitutes the complete STRP teleoperation platform: the master glove captures operator intent, the slave ZWHAND executes manipulation, and the dedicated tactile process closes the bilateral loop with sub-30 ms feedback latency.

Table 2 itemizes the bill of materials of the STRP-Glove master interface, which constitutes the core hardware contribution of this work; consistent with prior haptic-glove studies [12,33], the cost comparison is performed at the glove level. For completeness, the acquisition cost of the complete platform is itemized as follows: the ZWHAND dexterous hand (≈USD 6950), the UR5 arm (≈USD 16,670), and four miniature six-axis F/T sensors (≈USD 830 each), which together with the host PC bring the total to approximately USD 30,000.

#### 2.3.2. Fingertip Six-Axis Force Sensor

**Figure 4:**
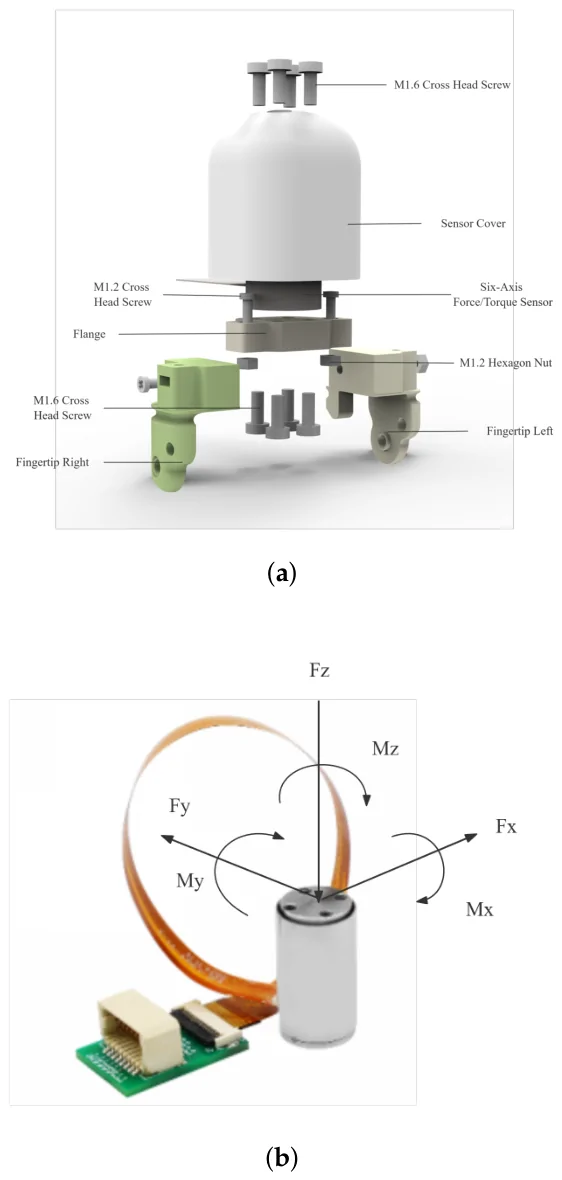
(**a**) Exploded view of the dexterous fingertip. (**b**) Assembled view of the miniature six-axis F/T sensor, illustrating the defined coordinate system, the applied forces (Fx, Fy, Fz) and moments (Mx, My, Mz) at the loading center.

The four-finger fingertip sensing unit is illustrated schematically in Figure 4. A custom miniature six-axis force/torque sensor [40], co-developed with the sensor manufacturer, is integrated at the distal tip of each finger to acquire real-time three-axis force and three-axis torque components. The sensor is mounted to the phalanx through a rigid fixture fabricated from SLA high-toughness resin. The assembly consists of a hemispherical protective shell, a sensor flange, and left/right fingertip bases. The flange rigidly couples the sensor body to the fingertip base, while the hemispherical shell is fastened to the top of the sensor via screws. This design ensures both robust mechanical integration and a compact footprint. More importantly, it creates a continuous force-sensing envelope: the hemispherical top surface together with the 360° cylindrical side wall of the shell act as effective contact interfaces. Consequently, contact forces and moments originating from arbitrary spatial directions can be accurately detected, furnishing multi-dimensional force feedback for dexterous grasping and complex environment interaction. The sensor has a diameter of 9.5 mm, with measurement ranges of ±50 N for tangential forces ($F_{x},F_{y}$), ±150 N for normal force ($F_{z}$), ±0.5 N·m for bending torques ($M_{x},M_{y}$), and ±1 N·m for torsional torque ($M_{z}$), and communicates with the slave controller through an I^2^C interface.

To eliminate the influence of different units, the data were first normalized according to Equation (6).

(6) $$Full\;Scale\;Output\left(\%F.S.\right)=\frac{Reading-Zero\;Reading}{Full\;Scale\;Span},$$

The various parameters of the six-axis force sensor are shown in Table 3.

While the current directional encoding algorithm primarily exploits the tangential force components ($F_{x}$, $F_{y}$) for real-time vibrotactile rendering, the six-axis measurement capability provides additional torque channels ($M_{x}$, $M_{y}$, $M_{z}$) that are reserved for future extensions. Specifically, the torque components can be leveraged for contact-point localization on the hemispherical fingertip shell via the moment–force ratio, as well as for detecting incipient slip or torsional motion during grasping—functionalities that are beyond the scope of the present work but represent natural extensions of the STRP platform.

## 3. Results

This section presents the experimental validation of the STRP teleoperation system. Section 3.1 verifies master–slave kinematic fidelity through static gesture reproduction. Section 3.2 validates the four-quadrant vibrotactile encoding via directional tactile discrimination. Section 3.3 reports teleoperated pick-and-place with grasp force modulation under varying feedback conditions. Section 3.4 quantifies the functional benefit of pose-independent tactile cues in visually occluded wall exploration. Section 3.5 assesses whether directional feedback supports functional manipulation beyond free-space probing.

All experiments reported in this section were performed by a single trained operator (male, 25 years, right-handed, with 6 h of prior teleoperation experience); this proof-of-concept design isolates the rendering capability of the hardware from inter-subject variability, and a multi-participant user study is identified as future work (Section 4).

### 3.1. Validation of Master–Slave Kinematic Mapping

**Figure 5:**
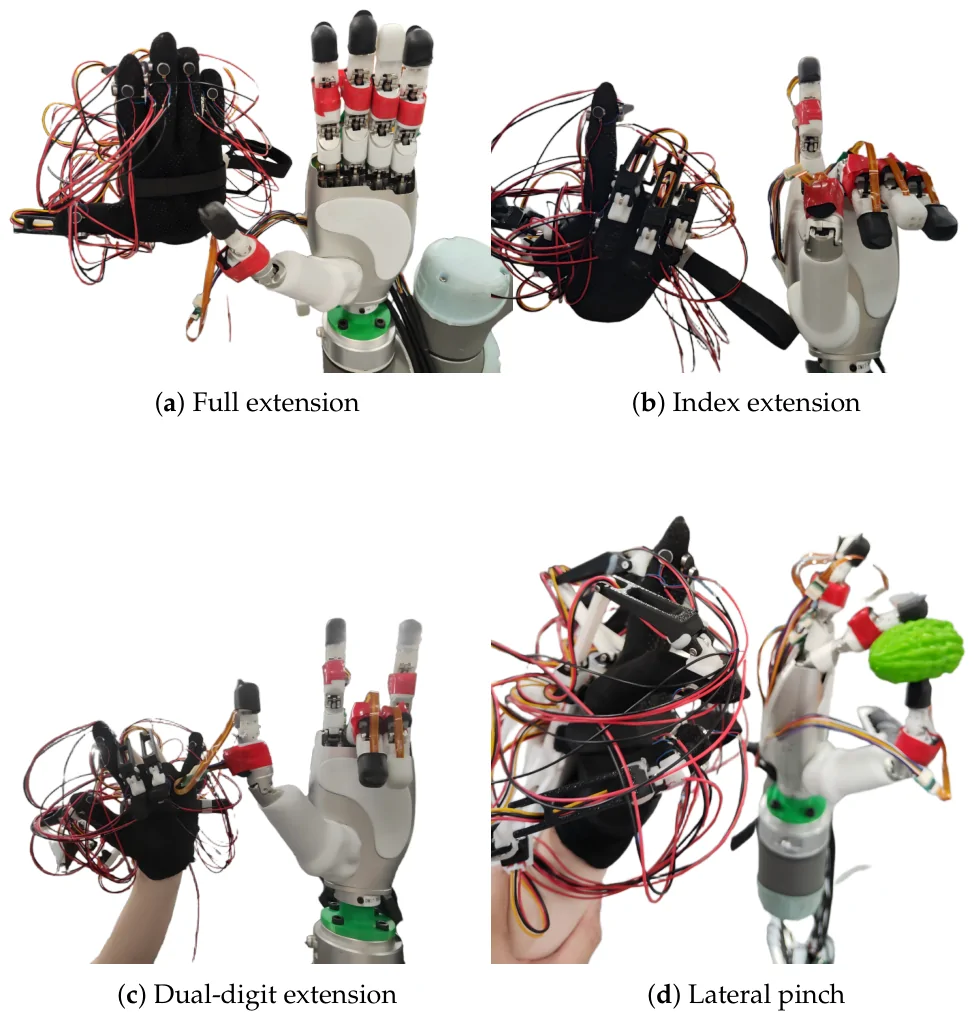
Qualitative comparison of static gesture reproduction. (**a**–**d**) The operator’s hand posture (left) and the corresponding ZWHAND slave response (right) viewed from the dorsal aspect. (**a**) Full extension. (**b**) Index extension with concurrent flexion of the remaining digits. (**c**) Dual-digit extension (index and little finger extended, middle and ring fingers flexed). (**d**) Lateral pinch (thumb–index pad contact).

To verify the kinematic consistency between the 15-DoF STRP-Glove and the 17-DoF ZWHAND, the operator performed four canonical postures: full finger extension, index extension with concurrent flexion of the remaining digits, dual-digit extension (index and little finger extended, middle and ring fingers flexed), and lateral pinch (thumb–index pad contact). The linkage-based exoskeleton reconstructed all target postures, exhibiting neither joint inversion nor coupling error, as shown in Figure 5. The end-to-end latency from master motion capture to slave actuation was approximately 28 ms.

### 3.2. Validation of Four-Directional Tactile Encoding

**Figure 6:**
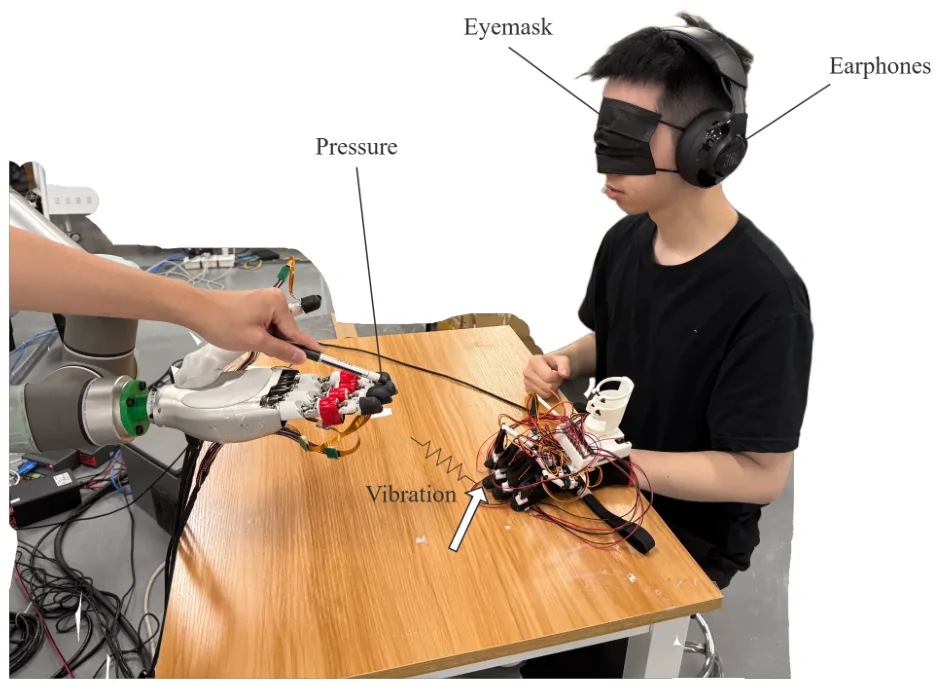
Experimental scene of the blind discrimination test. The operator, visually occluded and auditorily isolated, wears the STRP-Glove. An experimenter stimulates the slave ZWHAND fingertip with a blunt probe to trigger directional tactile cues.

To validate whether the four-directional tactile cues (left, right, dorsal, volar) rendered by the fingertip LRA array could be reliably perceived, a blind discrimination test was conducted. The operator wore the STRP-Glove with complete visual occlusion (eyemask) and auditory isolation (earphones), seated at a table with the master glove and the slave ZWHAND positioned within arm’s reach (Figure 6). Controlled normal forces (2–5 N) were applied to the slave fingertip via a blunt probe, triggering the corresponding directional vibration on the master glove through the real-time force–tactile mapping. The participant reported the perceived direction verbally. Each directional cue was presented 20 times (N=20 per direction). Before data collection, the operator completed a 10 min familiarization session in which each cue was presented with verbal confirmation until 6 consecutive correct identifications were achieved. The presentation order was randomized across trials.

The discrimination accuracy exceeded 95% for all four directions (Table 4). Errors were limited to occasional confusion between the dorsal and volar cues, attributable to the closer spatial proximity of the dorsal and volar LRAs on the fingertip pad compared with the lateral separation.

A supplementary test evaluated the four composite oblique cues (dorsal–left, dorsal–right, volar–left, volar–right), rendered through simultaneous superposition of the corresponding quadrant LRA pairs (Section 2.2.2). The four cues were presented in randomized order over 20 trials under the same blind protocol, with the four composite cues as the response alternatives (four-alternative forced choice, 25% chance level). A total of 17 of the 20 trials (85%) were correctly identified (Table 4), confirming that composite directional cues produced natively by channel superposition are reliably discriminated. The residual errors are attributable to confusion along the dorsal–volar axis, consistent with the error pattern observed for the cardinal cues; this can be mitigated by increased actuator spacing or a mid-line LRA (Section 4.1).

### 3.3. Teleoperated Pick-and-Place and Grasp Force Modulation

#### 3.3.1. Experimental Setup and Trajectory Tracking

The operator teleoperated the ZWHAND to grasp a glass beaker from a source table and place it into a designated target zone. Two feedback conditions were tested in randomized order: multi-directional tactile feedback (MTF) and no haptic feedback (NHF). Each condition comprised 15 trials (N=30 total). The target object was a 250 mL borosilicate glass beaker (outer diameter 70 mm, height 97 mm, smooth glazed surface). The operator was the same trained participant as in Section 3.2. Results are reported as mean ± SD together with the full distribution; given the single-operator proof-of-concept design, descriptive statistics are provided and inferential testing across subjects is deferred to the planned multi-participant study.

**Figure 7:**
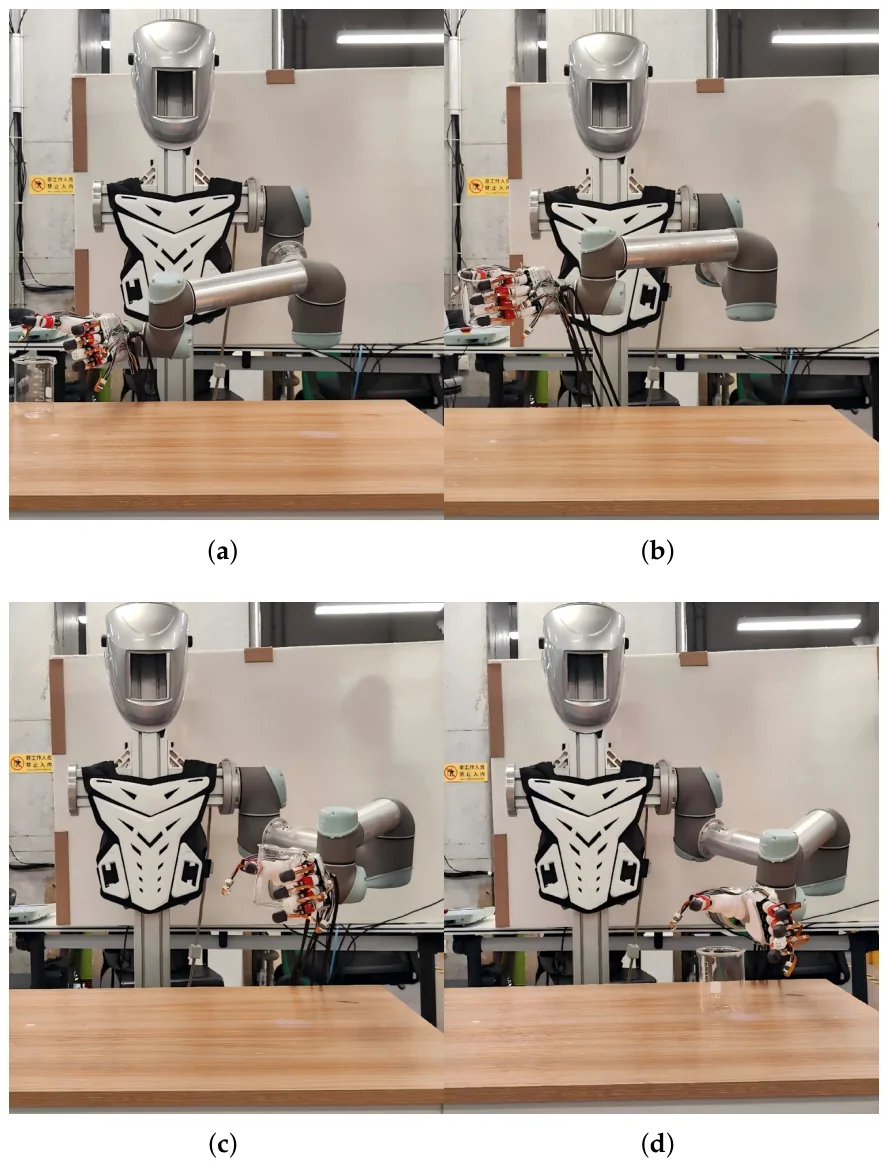
Qualitative demonstration of teleoperated beaker grasping. (**a**–**d**) The ZWHAND mounted on the UR5 robotic arm during a dynamic pick-and-place sequence. (**a**) Approach: the dexterous hand opens and positions above the beaker. (**b**) Lift-off: the beaker is raised from the table surface. (**c**) Transfer: the beaker is repositioned to the target location. (**d**) Release and withdrawal: the beaker is placed back on the table and the hand retracts.

Figure 7 presents a keyframe sequence of the complete teleoperated grasp–lift–transfer–place cycle under MTF, illustrating the four canonical phases of the pick-and-place task.

#### 3.3.2. Grasp Force Modulation

**Figure 8:**
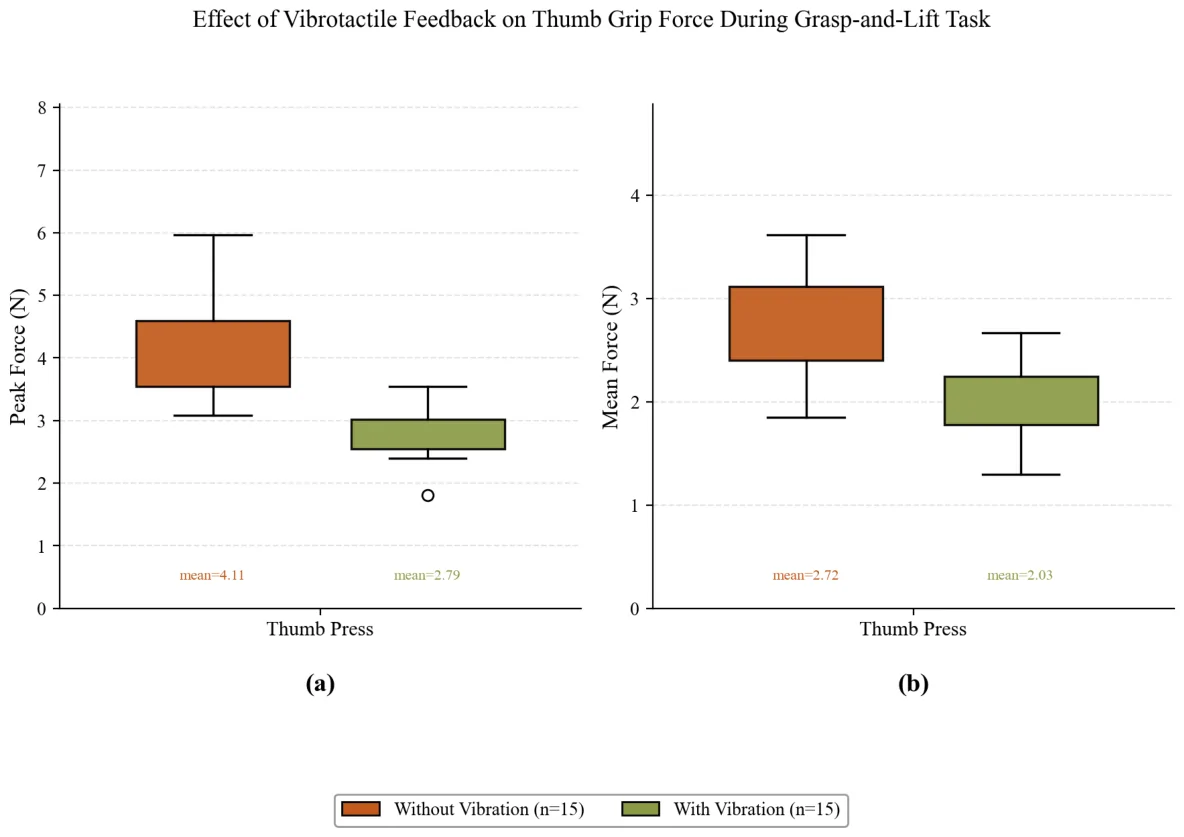
Grasp force modulation during the pick-and-place task. (**a**) Peak grip force. (**b**) Mean grip force. Box plots indicate median (horizontal line), interquartile range (box), and 1.5 × IQR whiskers. The mean value for each condition is annotated below the plot.

The fingertip six-axis force/torque sensor recorded the normal contact force $F_{z}$ throughout each trial. Figure 8 displays the aggregated results as box-and-whisker plots. Under NHF, the median peak grip force was 3.96 N (mean 4.11 N), with an interquartile range (IQR) spanning approximately 3.5–4.6 N. Under MTF, the median peak force dropped to 2.84 N (mean 2.79 N), representing a 32% reduction. Similarly, the mean grip force decreased from 2.72 N (NHF) to 2.03 N (MTF). These results demonstrate that directional tactile cues enable real-time force modulation, preventing excessive grip force caused by the absence of tactile feedback.

To quantify the functional benefit of multi-directional tactile feedback in a goal-directed manipulation task, we measured five performance metrics across the two feedback conditions (Table 5). Task completion time was defined as the interval from the operator’s first intentional motion command (i.e., opening the hand to pre-shape the grasp) to the moment the beaker was stably released inside the target zone. Peak grip force denotes the maximum normal contact force $F_{z}$ recorded at any fingertip during the lift–hold–transfer phase, reflecting the worst-case overload risk to fragile objects. Mean grip force was averaged over the entire contact duration, indicating sustained grasp effort. Slip incidents were counted whenever the beaker’s vertical position dropped by >2 mm after initial lift-off, signifying a loss of frictional contact. Success rate required the beaker to be placed upright within the target zone without tipping, cracking, or falling.

Multi-directional tactile feedback yielded improvements across all five metrics (Table 5). The 41% reduction in task completion time (from 31.2±4.5 s to 18.4±2.1 s) reflects more confident and decisive motion planning when the operator could perceive contact direction rather than relying on visual confirmation alone. The concurrent 32% drop in peak grip force and 25% drop in mean grip force indicate that directional cues allowed the operator to maintain a lighter, more adaptive grasp, eliminating the “grip-then-hold” strategy typical of no-feedback teleoperation. The near-elimination of slip incidents (from 1.5±0.5 to 0.2±0.1 per trial) further confirms that the reduced force did not compromise grasp stability; rather, the operator modulated force closer to the actual friction limit. Finally, the success rate rose from 73.3% (11/15) to 93.3% (14/15), with most NHF failures attributable to either excessive force cracking the beaker rim or insufficient force causing mid-transfer slippage—both error modes mitigated by real-time directional haptic awareness.

### 3.4. Visually Occluded Wall Exploration

**Figure 9:**
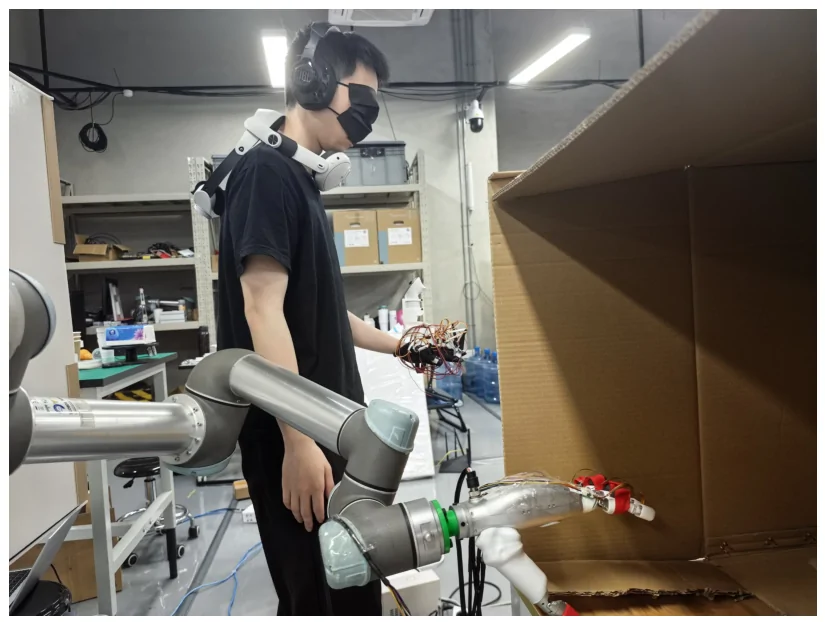
Experimental setup for visually occluded wall exploration. The operator, deprived of visual feedback and wearing an eyemask, teleoperates the ZWHAND inside the opaque enclosure. The STRP-Glove renders directional tactile cues according to the contact force vector measured at the slave fingertip.

To evaluate pose-independent exploration under visual deprivation, a wall-exploration task was conducted inside an opaque cardboard enclosure (0.6 m × 0.8 m base, 0.6 m in height). The operator, wearing an eyemask and the STRP-Glove for complete visual and auditory isolation, teleoperated the UR5 arm to sweep the ZWHAND fingertip across the interior surfaces and identify the positions of the target walls (Figure 9). Three feedback conditions were tested in counterbalanced order: multi-directional tactile feedback (MTF), Palm-Only (volar LRA only, no directional information), and no haptic feedback (NHF). To ensure statistical robustness, MTF and Palm-Only were evaluated over N=20 trials each; NHF served as a baseline with N=20 trials.

**Figure 10:**
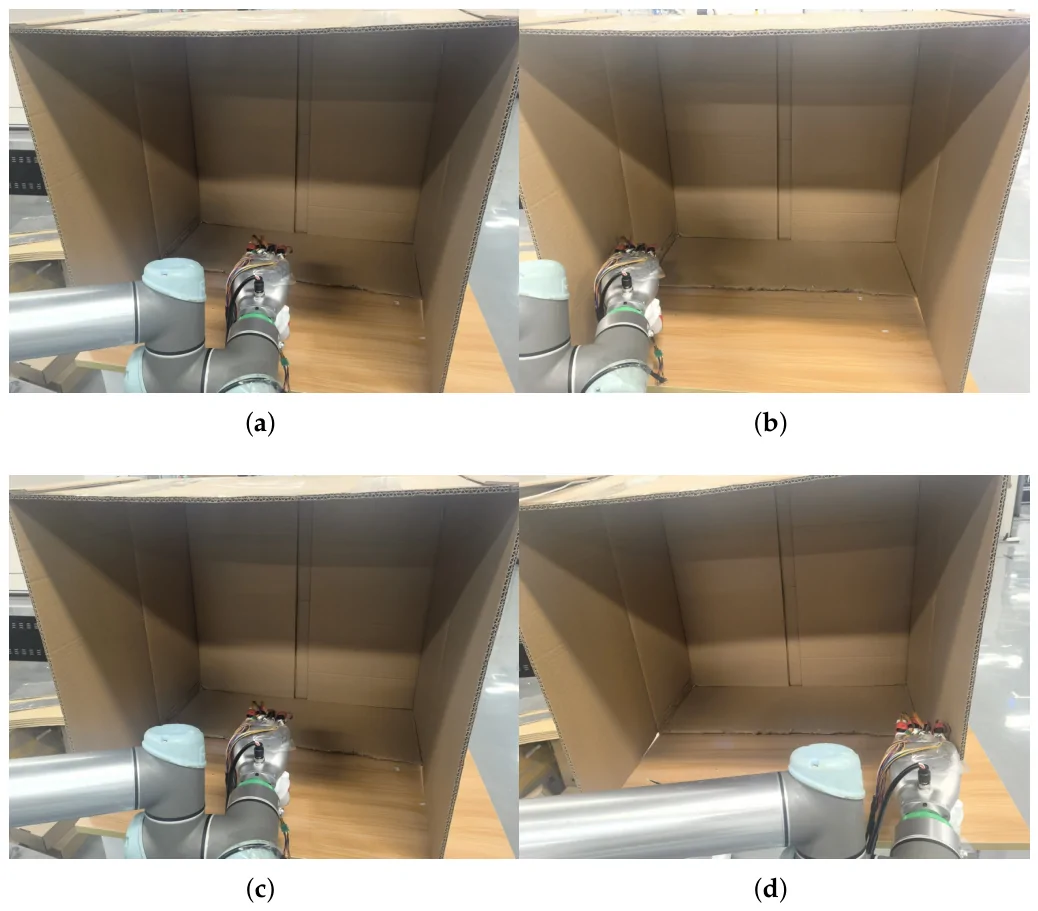
Visually occluded wall exploration demonstrating the systematic probing sequence. (**a**) The ZWHAND fingertip starts at the neutral center position. (**b**) The fingertip moves left and contacts the left wall, triggering left-directional vibrotactile feedback. (**c**) The fingertip returns to the neutral center before proceeding to the opposite side. (**d**) The fingertip moves right and contacts the right wall, triggering right-directional vibrotactile feedback. Throughout the sequence, the operator relies solely on directional tactile cues without visual feedback.

Under MTF, the operator followed a systematic probing sequence: starting from a neutral center position, moving left to contact the left wall, returning to center, and then moving right to contact the right wall. Figure 10 illustrates this sequence inside the opaque enclosure.

Under MTF, the operator consistently located all target walls, employing a systematic exploration strategy with zero wrist reorientation (Table 6). Under Palm-Only, only volar contact produced detectable feedback, whereas dorsal and lateral contacts remained silent, preventing the operator from locating walls outside the volar sensing sector. The operator repeatedly identified the left wall (19/20 trials) yet detected the right wall in only four of twenty trials, leaving the wall set incomplete and resulting in repeated collisions. Under NHF, exploration degenerated into disordered search with frequent wall collisions, and no trial achieved task completion.

These results demonstrate that directional feedback is not merely beneficial but essential for spatial exploration under visual deprivation: MTF enabled complete wall localization in all trials with a mean completion time of 23.6 ± 7.2 s, whereas the absence of directional information (Palm-Only and NHF) led to task failure in 80–100% of attempts. The MTF completion time was markedly shorter than that of Palm-Only, and the success-rate difference between MTF (100%) and Palm-Only (20%) was substantial. The Palm-Only condition further reveals that single-site volar feedback is insufficient—it only supports detection of volar-facing surfaces, leaving lateral and dorsal contacts undetected and preventing systematic spatial reasoning.

### 3.5. Visually Occluded Object Placement

**Figure 11:**
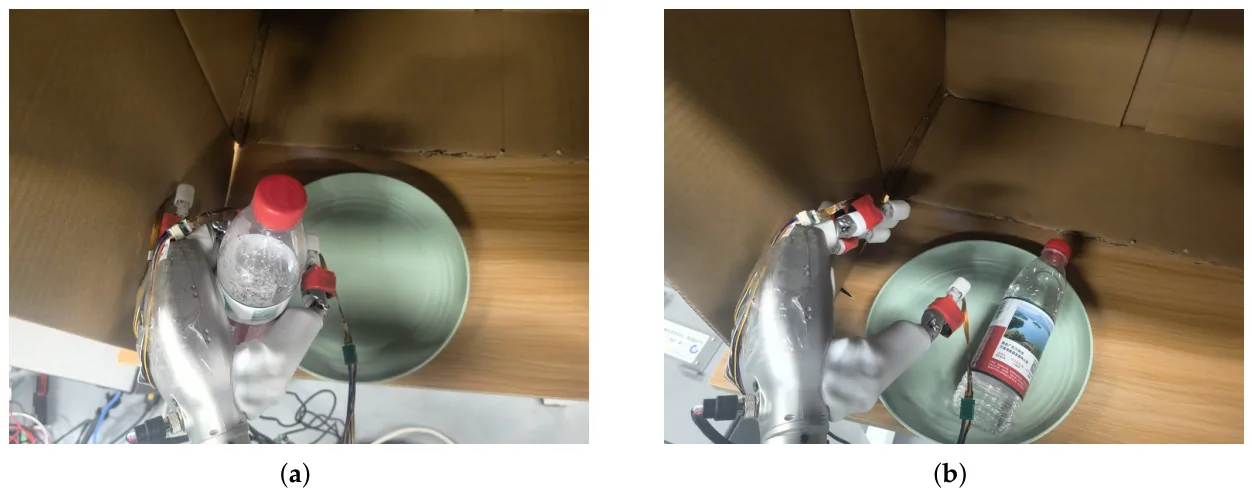
Visually occluded object placement. (**a**) The operator teleoperates the ZWHAND to transport the grasped bottle toward the right interior wall; fingertip contact triggers directional tactile cues that signal proximity to the boundary. (**b**) The bottle is released onto the target plate inside the opaque enclosure. Throughout the task, the operator relies solely on haptic feedback without visual confirmation.

To examine whether pose-independent directional cues support functional manipulation beyond free-space exploration, a visually occluded object-placement task was conducted inside the same opaque enclosure (Section 3.4). The operator began each trial with a cylindrical water bottle (height 227 mm, body diameter 64 mm, base diameter 52 mm) already grasped by the ZWHAND. Without visual feedback, the operator teleoperated the arm to translate the bottle horizontally toward the right-hand interior wall, detect contact via directional tactile cues, and release the object onto the floor-mounted target plate (Figure 11). A 30 s time limit was enforced. Three feedback conditions were tested in counterbalanced order: MTF, Palm-Only, and NHF (N=10 trials per condition).

Under MTF, right-directional vibration enabled the operator to perceive wall proximity and decelerate before impact; four of ten trials achieved successful placement at the target plate. The remaining six failures occurred during the release phase, mainly manifesting as the bottle slipping or tipping off the plate after detachment rather than colliding with the wall. It should be noted that in all MTF trials, the operator reliably identified the right-wall edge; failures did not stem from a lack of directional awareness, but from insufficient grip-force modulation during release.

Under Palm-Only and NHF, no trial achieved successful placement. Under Palm-Only, the single volar LRA channel could not convey external contact direction. In this task, the bottle was grasped by four fingers while the little finger was extended as the dedicated probing finger for wall exploration; the grasping and probing roles were thus assigned to different fingers, and no single channel had to render grip force and wall proximity at the same time. The volar pads of the four grasping fingers remained occupied by the bottle throughout the task, and wall contact was engaged exclusively on the dorsal or lateral aspects of the extended little finger, which never touched the bottle; the volar channel therefore carried no wall-contact signal—steady or transient—and the Palm-Only limitation is one of directional coverage rather than signal masking. The operator therefore lacked any tactile indication of bottle-to-wall proximity, resulting in continued pushing until collision. Under NHF, the complete absence of tactile feedback left the operator unable to estimate proximity to any surface; trials ended either with the object colliding with the wall or with the operator freezing in place until the time limit expired.

## 4. Discussion

### 4.1. Interpretation and Comparison

The experimental results validate the central design premise of STRP: pose-independent directional tactile cues are a distinct sensory modality that cannot be substituted by single-point vibration or vision alone. The four-quadrant LRA array achieved 97.5% discrimination accuracy, with the slight dorsal–volar asymmetry (95% vs. 100%) attributable to the compact fingertip anatomy; this could be mitigated in future hardware iterations by increasing the actuator spacing or adding a mid-line LRA.

In the pick-and-place task, MTF reduced peak grip force by 32% and improved task success compared with NHF. The critical advantage of the directional channel is that the operator knows where contact occurs, not merely that a contact exists—a distinction that magnitude-only feedback cannot provide. In the wall-exploration task, this advantage manifested as a stark contrast in completion rates: MTF located all target walls systematically, whereas Palm-Only and NHF failed to complete the task. The Palm-Only failure mode is particularly informative: the single volar LRA channel only responds to volar contact, leaving dorsal and lateral surfaces silent. Consequently, the operator could locate the left wall (volar contact) yet failed to detect the right wall (non-volar contact), directly illustrating why single-site feedback is insufficient for spatial exploration.

STRP thus occupies a unique position in the cost–capability landscape. Industrial systems (Shadow + HaptX) provide rich kinesthetic feedback at >USD 60,000 but lack spatial directional resolution. Low-cost alternatives (LEAP Hand, AnyTeleop) omit fingertip force sensing entirely. Recent haptic gloves (DOGlove, CDF-Glove) have integrated force feedback with dexterous hands, yet their rendering remains confined to magnitude or single-site vibration without explicit directional encoding. STRP bridges this gap at roughly one-fifth the hardware cost of comparable haptic gloves (e.g., DOGlove) and approximately two orders of magnitude below that of industrial kinesthetic systems (e.g., SenseGlove).

### 4.2. Limitations and Future Directions

The current system has been validated under controlled laboratory conditions with a trained operator; generalizability to novice users and diverse hand morphologies remains to be assessed.

Vibrotactile feedback also carries perceptual constraints. In the present study, individual sessions were kept within approximately 30 min with rest intervals, within which no fatigue-related degradation was observed and all tasks were completed reliably. Under substantially longer continuous operation, however, sensory adaptation may attenuate perceived intensity and operator fatigue may become a limiting factor; systematic evaluation of prolonged-use performance will be addressed in future work.

Regarding mechanical durability, no measurable calibration drift was observed over the study period, as the per-session calibration re-records the sensing extrema; nevertheless, long-term wear of the 3D-printed PLA revolute joints and the SLA resin sensor fixtures remains uncharacterized. Future hardware iterations will migrate load-bearing links to fiber-reinforced filament (e.g., PPA-CF) with metal pin joints, accompanied by accelerated life-cycle testing. Dynamic characterization of the fingertip six-axis sensors under continuous sliding contact—including dynamic crosstalk and zero-drift behavior—will likewise be pursued.

The object-placement task exposed a bottleneck in force–release coordination. Notably, in all MTF trials, the operator localized the right wall correctly; every failure occurred after detachment, as the bottle slipped or tipped off the plate. The limiting factor is therefore no longer directional perception but the open-loop execution of the release trajectory: precise grading of force reduction from vibrotactile cues alone is demanding for human operators. Addressing this bottleneck requires dedicated algorithmic treatment beyond the scope of the present work. In future iterations, the three-axis torque channels of the fingertip sensors—currently unused in the rendering loop—will be exploited by the slave-side controller to detect moment shifts and incipient slip during release, enabling local reflexes such as re-grasping or slowing finger opening while the operator retains directional authority. The systematic design and experimental validation of such slip-prevention strategies constitute an important direction of our future work.

Future work will pursue three directions. Algorithm: implement the slave-side slip-detection and release-assist reflexes described above. Hardware: increase LRA spacing or add a mid-line actuator to improve dorsal–volar discrimination. Evaluation: conduct user studies with a broader participant pool (including novices) and extend tasks to liquid-filled beakers, energized components, and nuclear-decommissioning mock-ups to assess ecological validity.

## 5. Conclusions

This paper presented STRP, a low-cost teleoperation system with concurrent motion capture and directional haptic feedback that encodes fingertip contact force vectors into four-directional vibrotactile cues. The key contributions are as follows: (i) a wearable 15-DoF exoskeleton glove with radially magnetized Hall-effect sensing and a 16-channel LRA array; (ii) a directional encoding algorithm mapping six-axis force data to spatially resolved tactile feedback with approximately 28 ms end-to-end latency; (iii) experimental validation demonstrating that pose-independent directional perception shortens task completion time, reduces peak grip force, and enables wall exploration impossible under single-site or no-feedback conditions.

The results support the thesis that spatial directional tactile cues constitute a distinct sensory modality, one that differs from conventional single-amplitude vibration in conveying the location of contact rather than only its occurrence, and that this modality is necessary for visually occluded dexterous manipulation.

Several limitations bound the present claims. The evaluation relied on a single trained operator under controlled laboratory conditions. The force–release coordination bottleneck discussed in Section 4 remains to be addressed by slave-side assistive algorithms. Finally, long-term mechanical durability and perceptual adaptation under prolonged use await systematic characterization. These points define the immediate agenda of our future work.

STRP establishes a hardware and algorithmic foundation for affordable, high-fidelity haptic teleoperation in hazardous environments.

## Figures and Tables

**Table 1:** STRP overall performance parameters.

Items	Parameters
Glove materials	PLA
Fingertip material	High toughness resin (Somos® LEDO)
LRA	LD0825BC-0169F
Hall-effect sensor	OH49E
Measurable DoF	15
Coupled computation DoF	5
Force feedback latency to hand	≈28 ms
Communication protocol	RS485 & Modbus-RTU

**Table 2:** Cost breakdown of the STRP-Glove master interface.

Type	Item	Num	Price (USD)
Electronic components	Main control board (STM32F103)	1	67.92
	Hall sensors (OH49E)	15	2.08
	LRAs (LD0825BC-0169F)	16	20.00
	Nd-Fe-B magnets (N35)	15	1.29
Non-standard parts	3D-printed parts (PLA)		3.00
	Resin fingertip bases		11.11
Other components	Glove fabric	1	1.67
	Adhesive tape	1	0.83
	Adhesive	1	2.22
	Screws		0.83
**Total cost**			**110.95**
DOGlove [33]			600.00
SenseGlove Nova 2 [12]			5999.00

Prices are converted from Chinese retail/component markets (JLCPCB, Taobao) at 1 USD = 7.2 CNY.

**Table 3:** Detailed specifications of the miniature six-axis F/T sensor.

Items	Parameters
Diameter	Φ 9.5 mm
Height	17 mm
Force range ($F_{x}$, $F_{y}$)	±50 N
Force range ($F_{z}$)	±150 N
Torque range ($M_{x}$, $M_{y}$)	±0.5 N·m
Torque range ($M_{z}$)	±1 N·m
Non-linearity	<0.4%F.S.
Hysteresis error	<0.8%F.S.
Repeatability error	<0.4%F.S.
Crosstalk error	<3%F.S.
Resolution	≈25 mN ($F_{x}$, $F_{y}$); ≈124 mN ($F_{z}$)

**Table 4:** Directional tactile discrimination results (N=20 presentations per direction). The last row reports the supplementary test with the four composite oblique cues.

Direction	Correct/Total	Accuracy (%)
Left	20/20	100
Right	20/20	100
Dorsal	19/20	95
Volar	19/20	95
**Overall**	**78/80**	**97.5**
Composite oblique cues	17/20	85

**Table 5:** Teleoperated pick-and-place task metrics (mean ± SD, N=15 per condition).

Metric	NHF	MTF
Task completion time (s)	31.2±4.5	18.4±2.1
Peak grip force (N)	4.11±0.9	2.79±0.5
Mean grip force (N)	2.72±0.6	2.03±0.4
Slip incidents per trial	1.5±0.5	0.2±0.1
Success rate (%)	73.3 (11/15)	93.3 (14/15)

**Table 6:** Occluded wall exploration performance (N=20 trials per condition).

Metric	MTF	Palm-Only	NHF
Task completion rate	20/20	4/20	0/20
Left-wall success	20/20	19/20	0/20
Right-wall success	20/20	4/20 ^*a*^	0/20
Completion time (s)	23.6 ± 7.2	40.4 ± 11.8 ^*b*^	— ^*c*^

^*a*^ Palm-Only renders feedback only for volar contact; right-wall approach primarily engages non-volar surfaces. ^*b*^ Mean ± SD across successful trials (n=4). ^*c*^ All trials failed.

## Data Availability

The data and original materials supporting the reported results are available within the article/Supplementary Materials. Supplementary inquiries or requests for additional details should be addressed to the corresponding author.

## Supplementary Materials

The following supporting information can be downloaded at https://www.mdpi.com/article/10.3390/biomimetics11090609/s1, Video S1: Demonstration of the STRP teleoperation platform with spatial force feedback.

## Abbreviations

The following abbreviations are used in this manuscript:

STRP	Spatial Teleoperation Research Platform
LRA	Linear resonant actuator
DoF	Degrees of freedom
F/T	Force/torque
MTF	Multi-directional tactile feedback
NHF	No haptic feedback
DIP	Distal interphalangeal
PIP	Proximal interphalangeal
MCP	Metacarpophalangeal
IP	Interphalangeal
TM	Trapeziometacarpal
PLA	Polylactic acid
